# Supplementary material for: Longitudinal genomic analysis of Neisseria gonorrhoeae transmission dynamics in Australia
Source: Nat Commun. 2024 Sep 14;15:8076. doi: 10.1038/s41467-024-52343-0 (PMC11401900; doi:10.1038/s41467-024-52343-0)
Supplement: Supplementary file 1 — Supplementary Information [file 41467_2024_52343_MOESM1_ESM.pdf]

Longitudinal Genomic Analysis of *Neisseria gonorrhoeae* Transmission Dynamics in Australia

Supplementary Appendix

Taouk et al.

Supplementary Figure 1

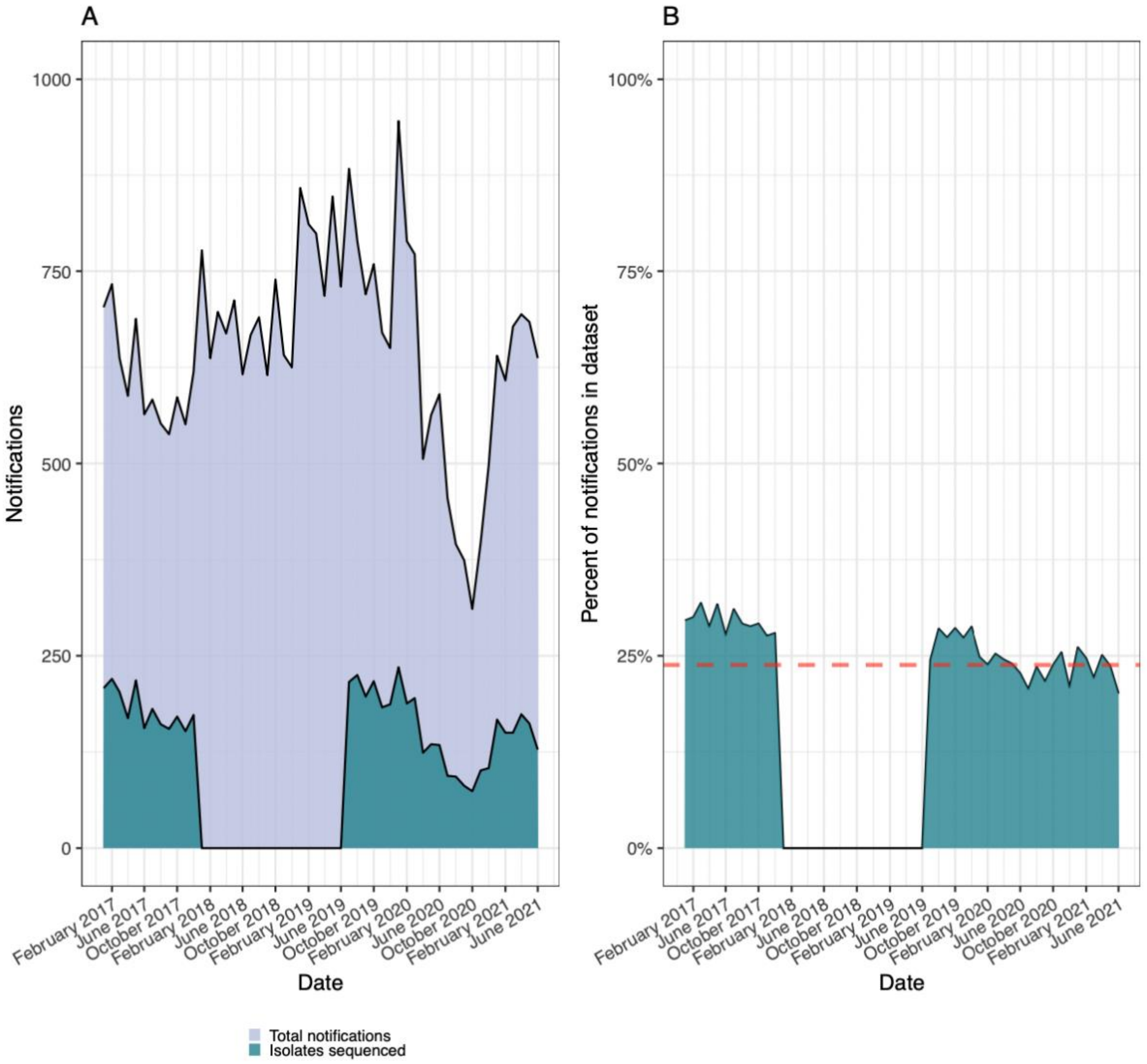

Number of notifications of *Neisseria gonorrhoeae* compared to number of isolates sequenced **A.** The number of gonorrhoea notification in Victoria received through the national Notifiable Diseases Surveillance System for each month between January 1st 2017 and June 30th 2021 compared to the number of isolates sequenced and included in this study. No samples collected between 1st January 2018 and 30th June 2019 were sequenced due to funding constraints. **B.** The percentage of notifications with associated sequences each month. The red dashed line represents the median percentage of notifications with associated sequences across the dataset (24%).

Supplementary Figure 2

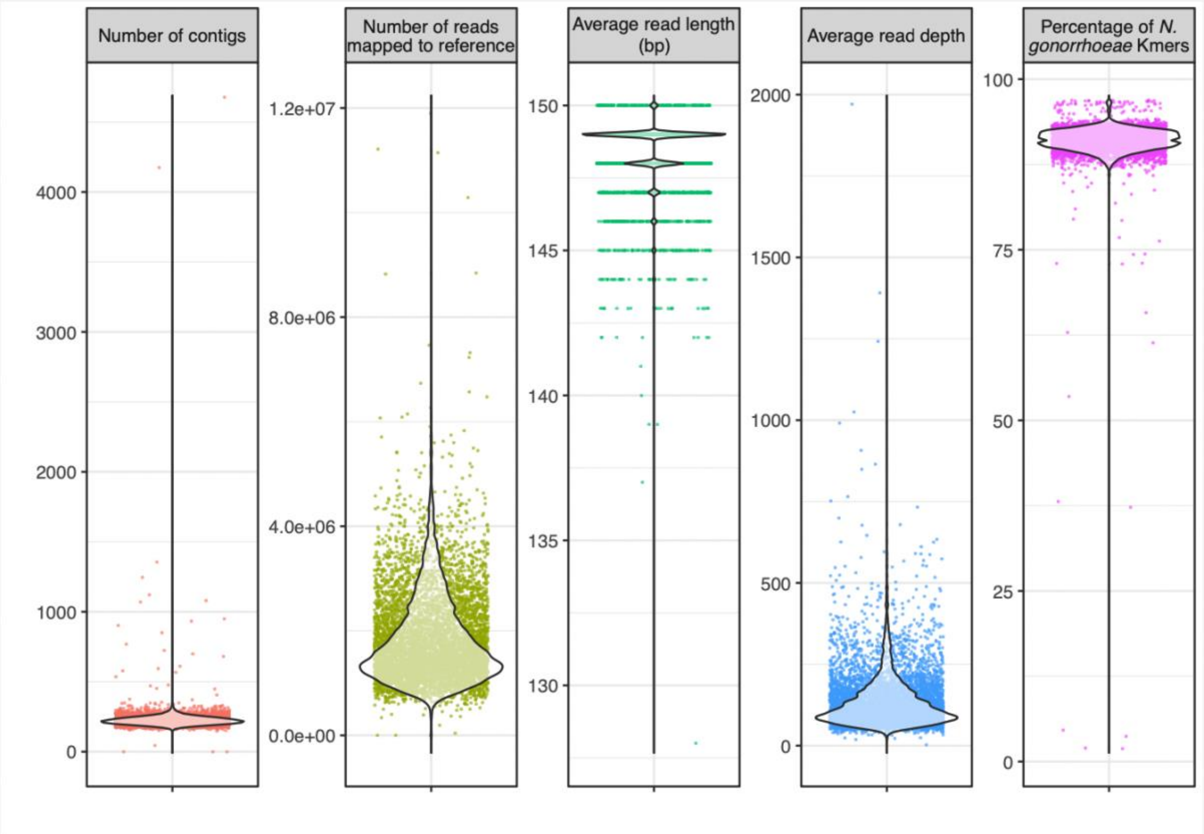

Quality control filtering outcomes for all 6,329 isolates sequenced. Of the 6,329 isolates sequenced, 8 were excluded due to an average sequencing depth of <30, and 85 were excluded due to assemblies of ≥300 or 0 contigs. 2 isolates were excluded as they were missing an accompanying date of collection, 2 were excluded as they were missing an accompanying age of individual, and 17 were excluded as they were collected from individuals under the age of 16. 6,215 isolates remained. Of these, 49 were duplicate colony picks and 285 were collected from the same individual on the same day at different body sites and were not included for clustering analyses. 5,881 isolates were included for clustering and downstream analyses.

Supplementary Figure 3

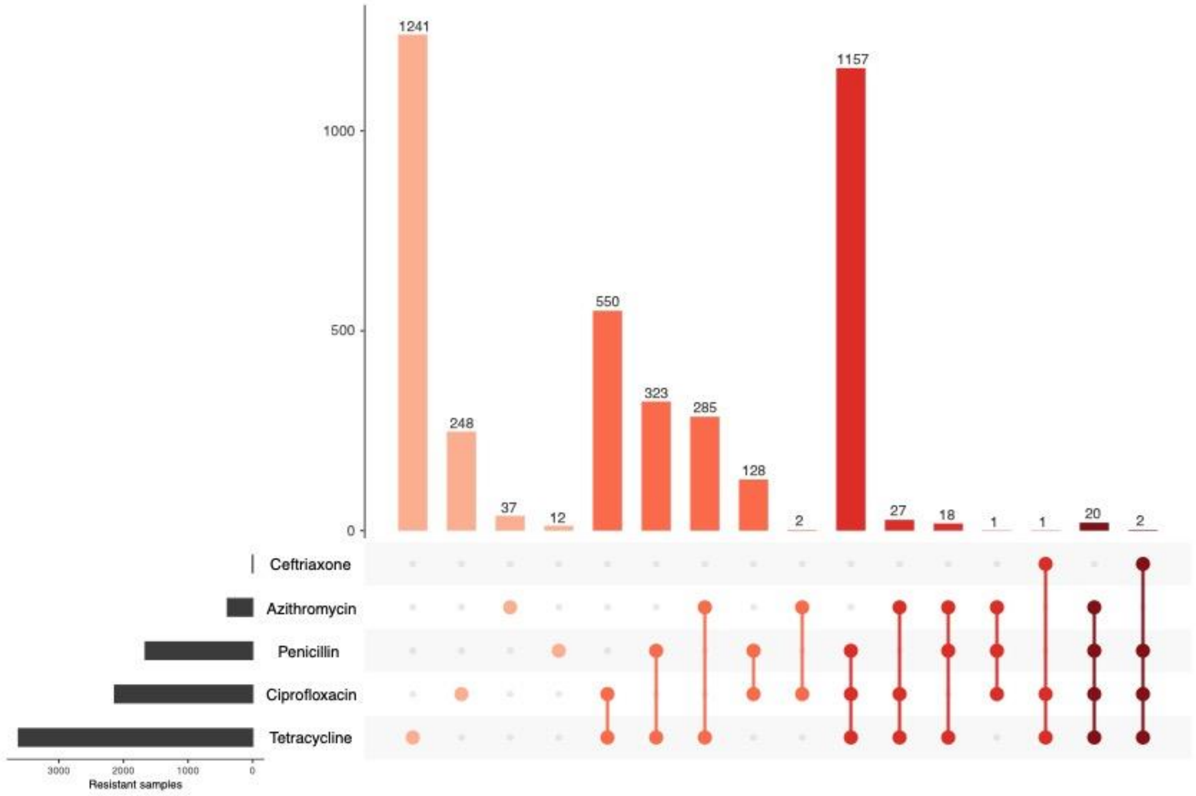

An upset plot representing phenotypic antimicrobial resistance. The primary axes of the plot are the antibiotics tested with each circle representing a distinct class of antibiotics, and intersections illustrating overlapping areas between circles to denote isolates resistant to multiple antibiotic classes. The number of isolates resistant to each class of antibiotics is represented by the bars on the left. The number of isolates resistant to each intersection of classes is represented by the bars on the top. Resistance to one antibiotic is coloured in the lightest pink, and so forth until the intersection of four classes is coloured in the darkest red. Decreased susceptibility to ceftriaxone is shown in place of resistance. There were no isolates resistant (or decreased susceptibility for ceftriaxone) to all five classes.

Supplementary Figure 4

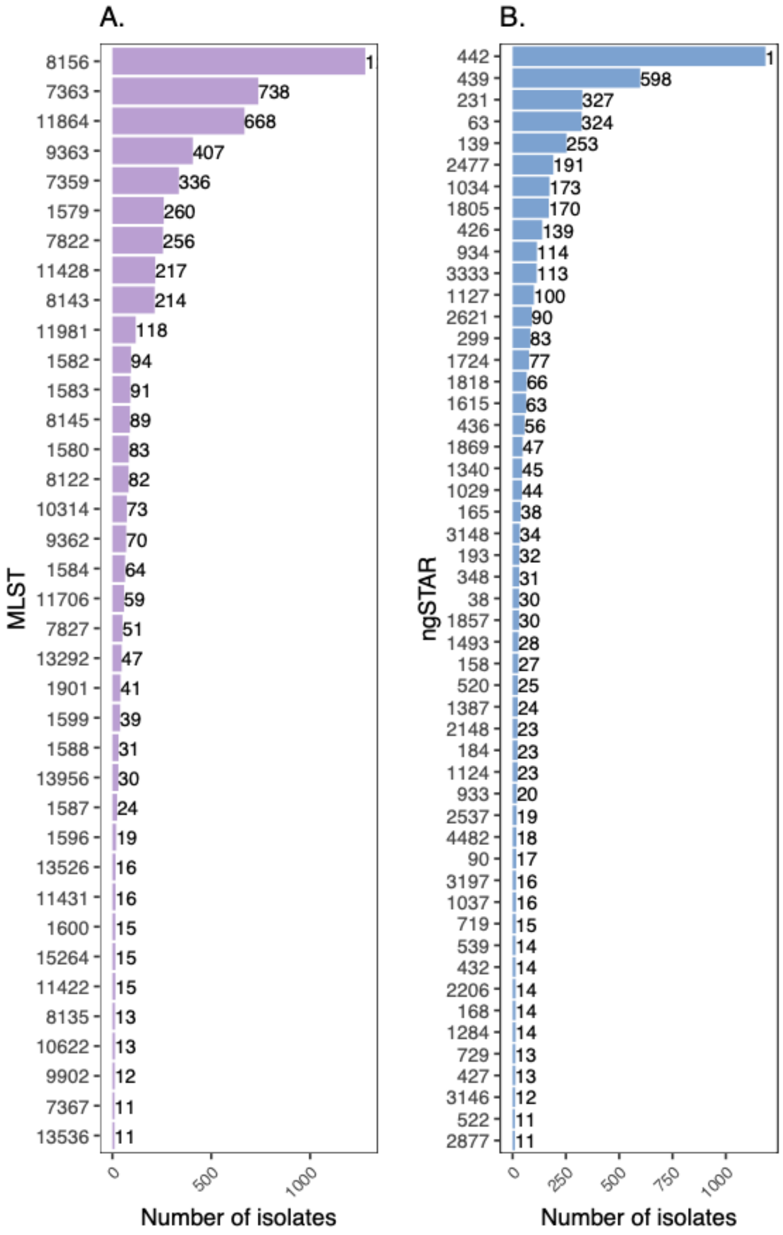

Number of isolates in each sequence type for **A.** MLST and **B. NG-STAR**. Only sequence types with >10 genomes are included.

Supplementary Figure 5

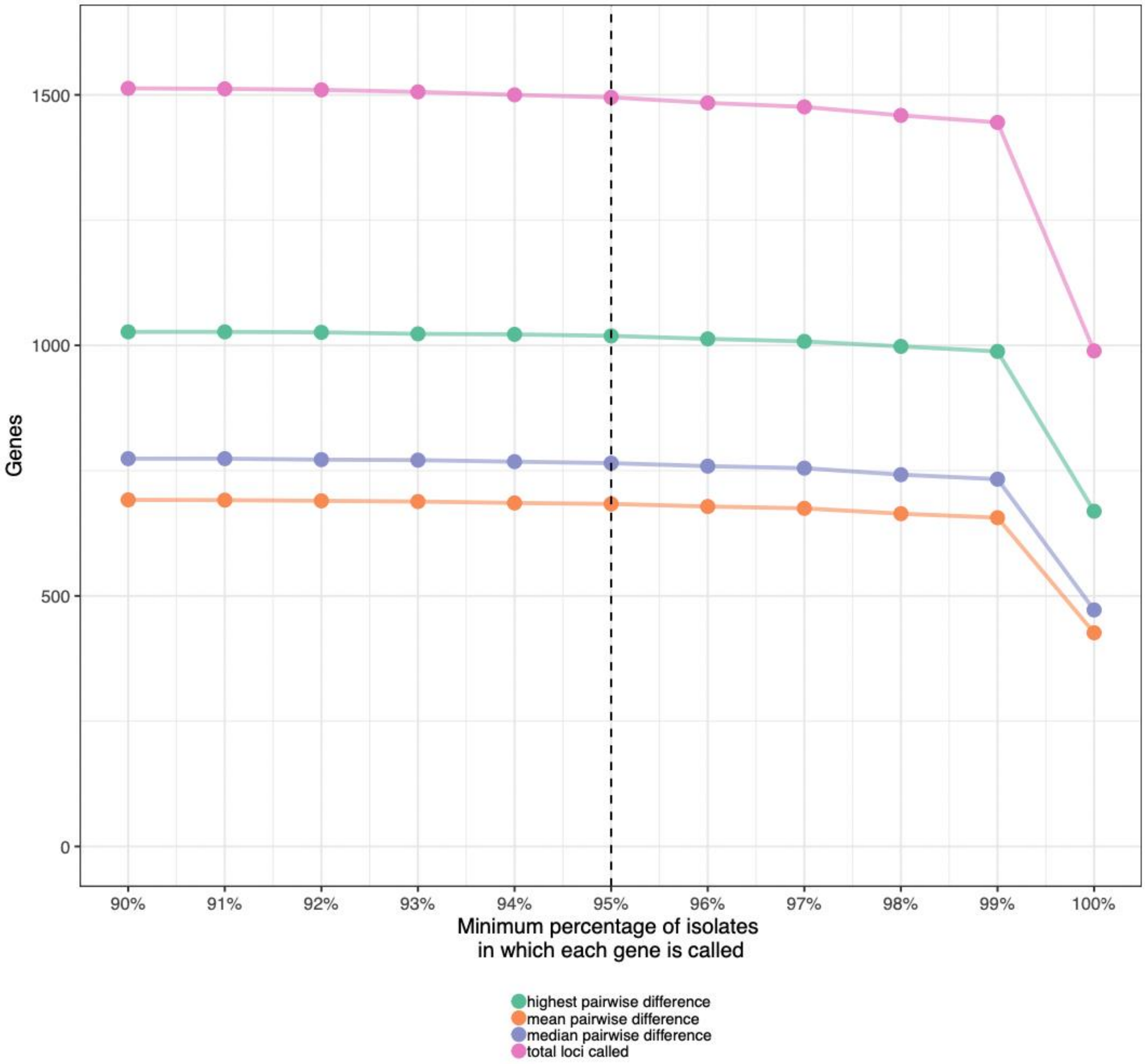

Number of genes included in the cgMLST scheme at various thresholds. The x axis represents the minimum percentage of isolates (out of the total 5,881 included in the study) that any gene must be successfully identified in to be included in the cgMLST scheme. To determine a threshold for inclusion, the cut off threshold was iterated for a range from 90% to 100%. The y axis represents the number of genes, with the pink line showing the total genes called at each iterated percentage cut off. The green, orange, and purple lines represent the highest, mean, and median pairwise differences within the dataset at each cut-off percentage.

Supplementary Figure 6

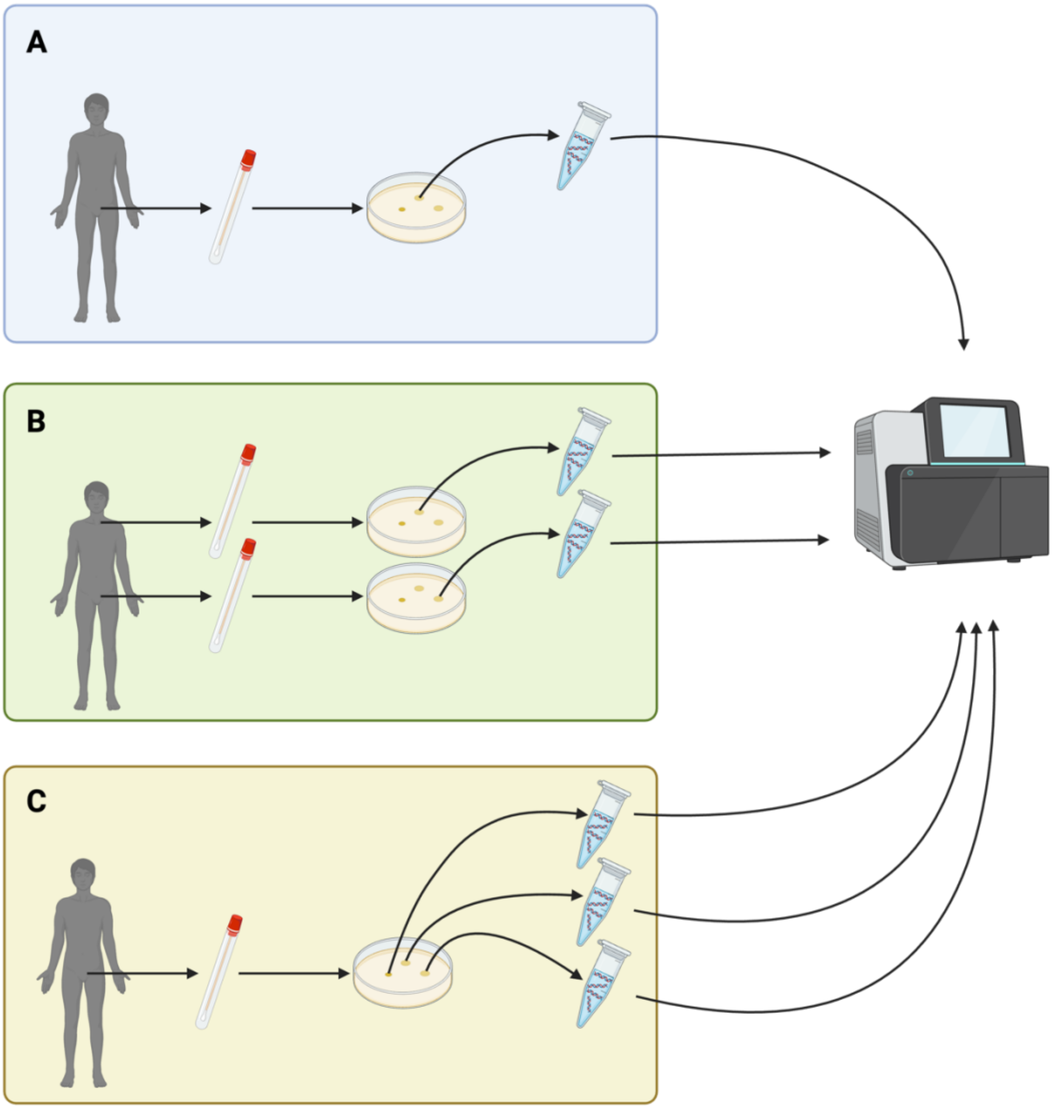

Schematic of calibration samples. **A.** Isolates collected from a single individual from one body site. **B.** Isolates collected from the same individual at two or more different body sites. **C.** Isolates collected from the same individual from the same body site and libraries generated for multiple colonies from the culture plate. Created with BioRender.com released under a Creative Commons Attribution-NonCommercial-NoDerivs 4.0 International license.

Supplementary Figure 7

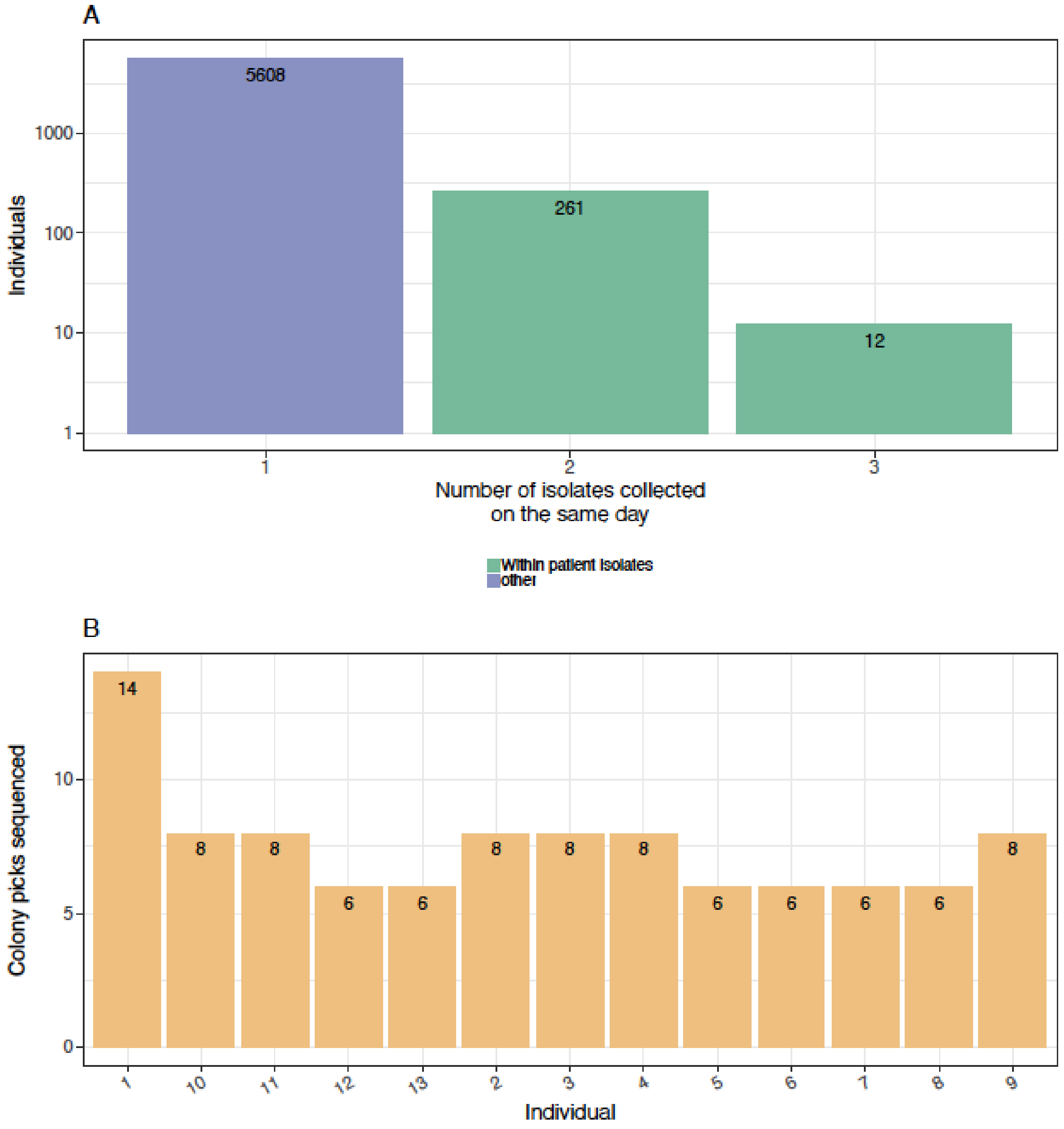

Summary of within-individual and within-site isolates **A.** The number of isolates sequenced from a single individual, where isolates were collected from different anatomical sites on the same day. **B.** The number of colonies sequenced for each isolate with multiple colony picks.

Supplementary Figure 8

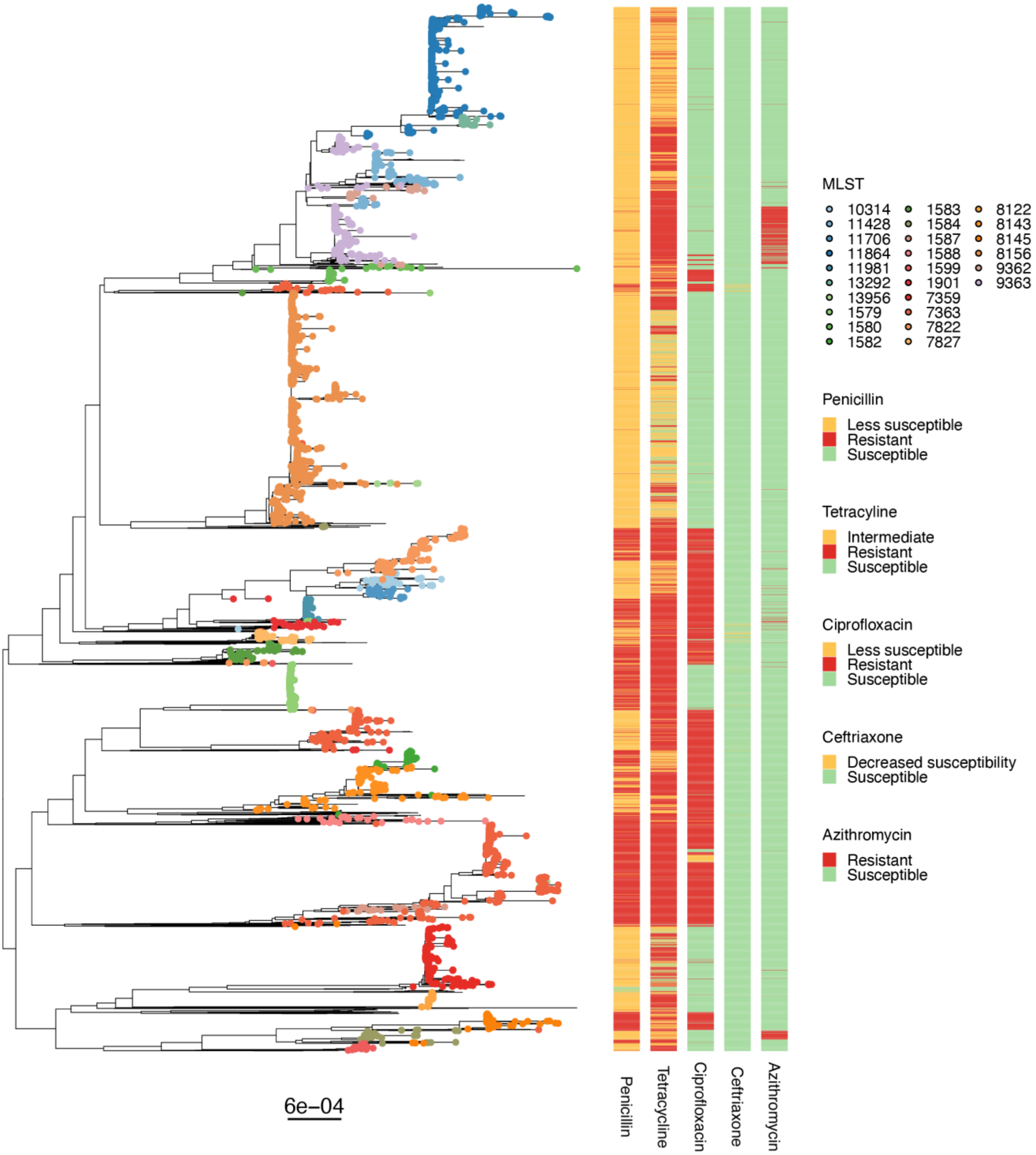

Population structure of 5,881 *N. gonorrhoeae* isolates included in this study. The mid-point rooted maximum-likelihood tree (derived from concatenated cgMLST alignment) is plotted on the left. Tips are coloured by MLST for MLST profiles which appear in at least 30 isolates. Phenotypic resistance profiles are shown on the right. The tree scale represents substitutions per site.

Supplementary Figure 9

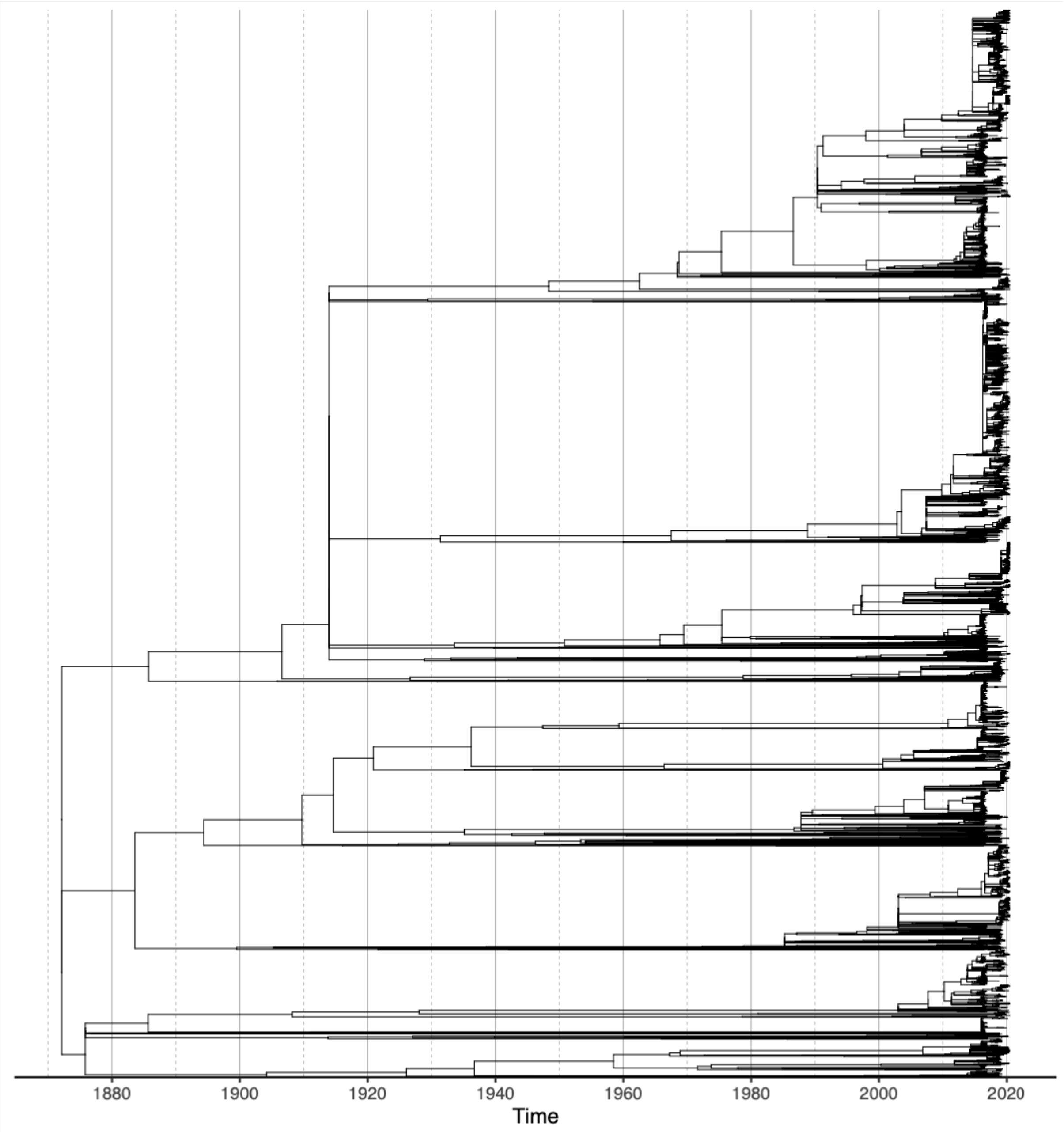

Ancestral reconstructed phylogeny of 5,881 *N. gonorrhoeae* isolates dated using least-squares dating (LSD). Tree rate: 2.399 x 10^{-5} , tMRCA: 1873.125327. The phylogenetic tree illustrates the evolutionary relationships among the dataset, with each branch representing genetic divergence events.

Supplementary Figure 10

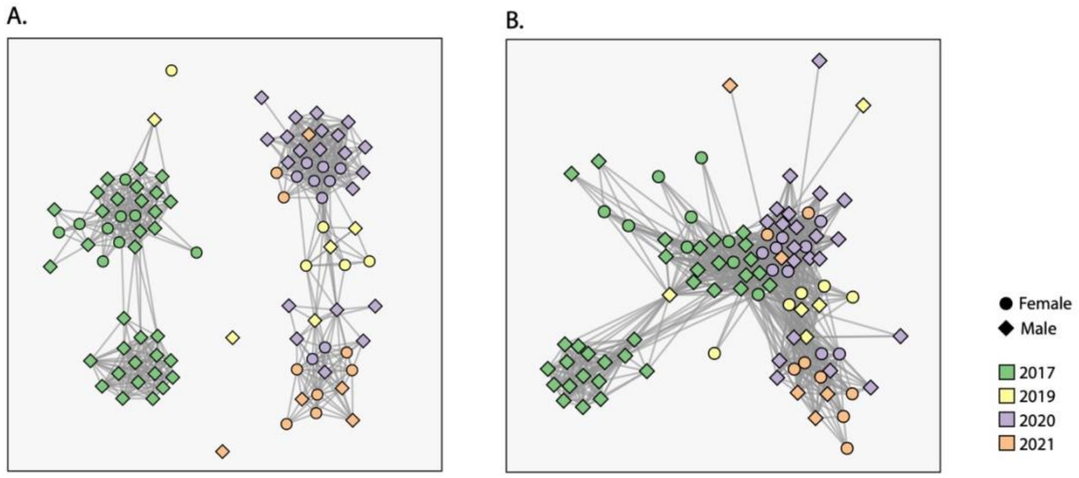

Genomic cluster 33 (n = 95 isolates), whereby **A.** using the unadjusted cgMLST pairwise allelic differences, the isolates are grouped into two clusters and 3 singletons and **B.** using the adjusted cgMLST pairwise allelic differences, the isolates form one transmission cluster. Isolates are coloured by year of collection to show that without adjusting for the collection date, isolates cluster by collection date. Isolates are shaped by sex of individual from which they were collected to show similar proportions of males and females in the two clusters in panel A; epidemiologically suggesting that this is a heterosexual associated cluster.

Supplementary Figure 11

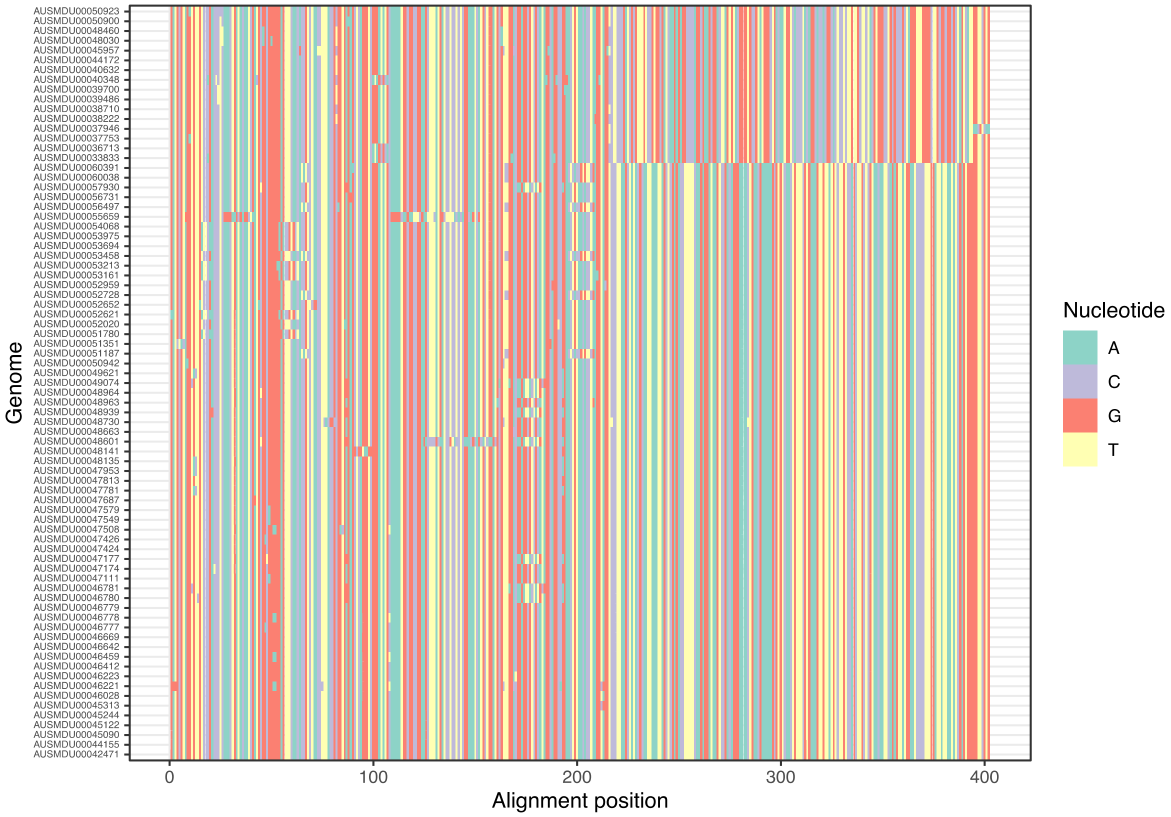

A SNP alignment for genomic cluster 230 showing that 16 isolates all share the same 177 SNPs (position 224 – 401). These SNPs can all be found within 4 consecutive genes in the genome (NGO_2109, NGO11390, NGO2111 and NGO2112). As all 16 isolates sharing SNPs in these genes were collected before June 2020, this may indicate a recombination event that occurred sometime before 2020, resulting in177 SNPs which would be treated as 177 individual evolutionary events, instead of a single recombination by SNP based phylogenetic methods. While cgMLST summarises these 177 SNPs into 4 allelic differences, highlighting the appropriateness of the cgMLST clustering method for this dataset.

Supplementary Figure 12

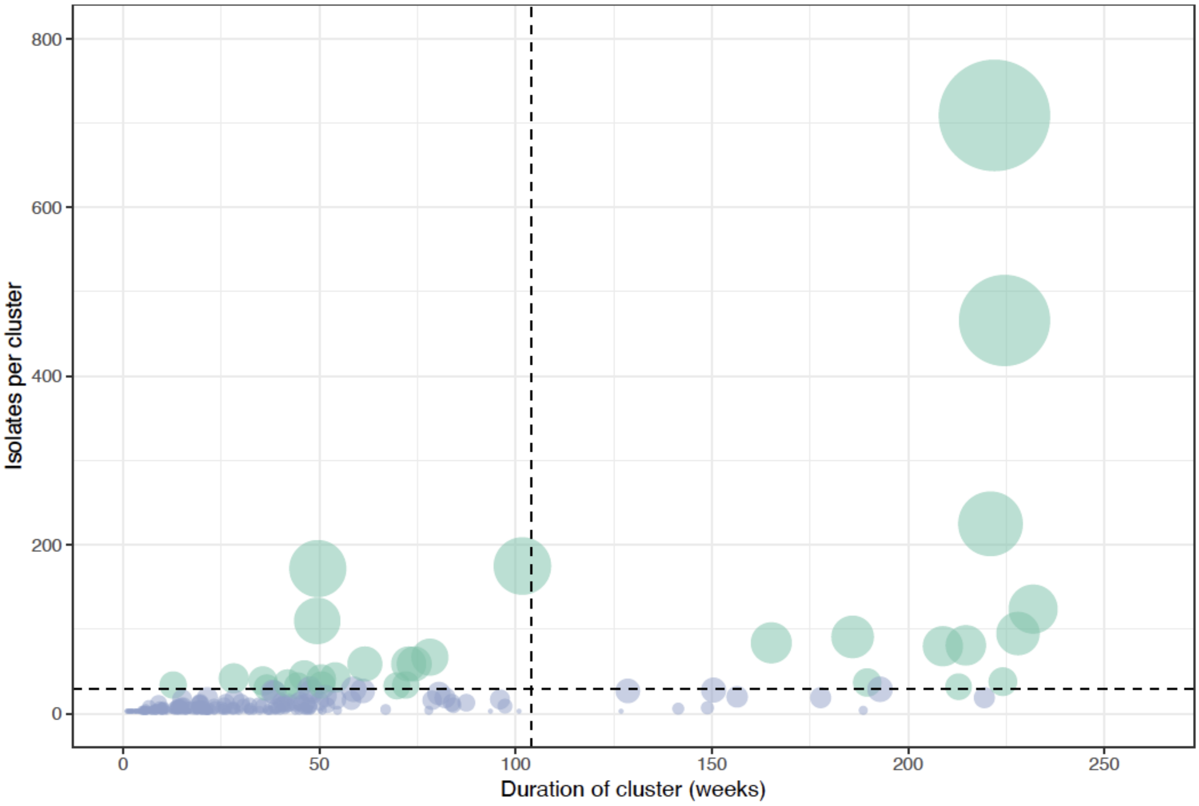

The number of isolates per cluster and the duration of each cluster as calculated in weeks from the earliest date of sample collection to the latest date of sample collection per cluster. Each cluster is represented by a circle and the size of the circle is proportional to the number of isolates per group. Groups are coloured by if they are large groups (green) or not (blue). The horizontal line is at 30, the threshold required for a cluster to be defined as "large". The vertical dotted line is at 104 weeks which was the threshold required for a cluster to be defined as "persistent".

Supplementary Figure 13

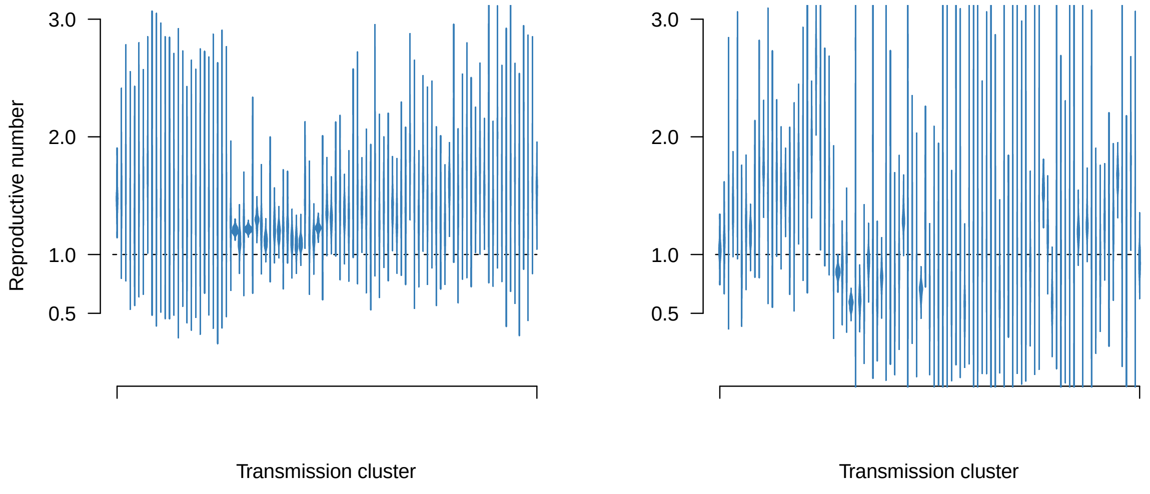

The estimated R_e for each cluster where **A.** shows the distribution of R_e values occurring before the modelled time slice for each of the 97 clusters and **B.** shows the distribution of R_e values occurring after the modelled time slice for each of the 97 clusters.

Supplementary Figure 14

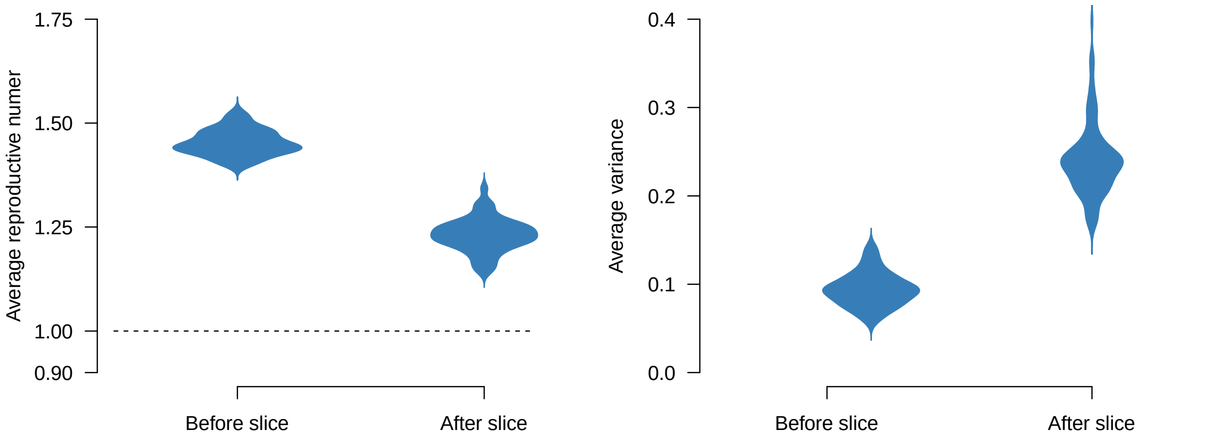

A violin plot of the **A.** arithmetic averages of the 97 clusters' R_e for and the **B.** arithmetic of the 97 clusters' R_e variance for clusters occurring before and after the modelled time slice.

Supplementary Figure 15

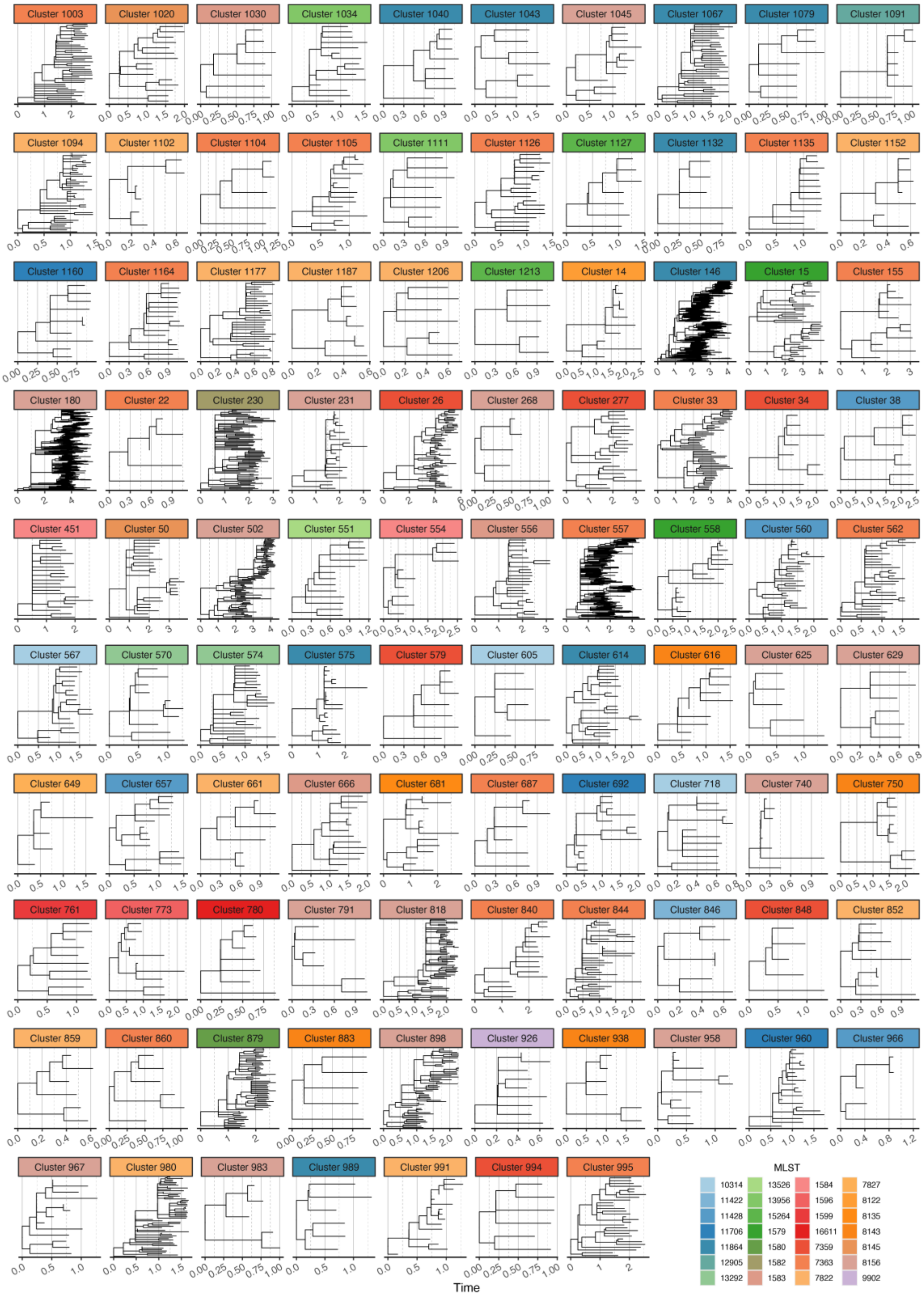

Ancestral reconstructed phylogenies for each of the cluster phylogenies generated using least-squares dating (LSD). Phylogenetic trees are labelled by MLST. The X axis is in years from the MRCA for each cluster.

Supplementary Table 1

	Susceptible	Less susceptible	Decreased susceptibility	Intermediate	Resistant
**Penicillin**	≤0.03	0.06-0.5			≥1
**Tetracycline**	≤0.25			0.5-1.0	≥2
**Ceftriaxone**	≤0.03		0.06-0.25		Not defined
**Ciprofloxacin**	≤0.03	0.06-0.5			≥1
**Azithromycin**	<1				≥1
**Spectinomycin**	≤64				>64

Interpretation of *Neisseria gonorrhoeae* MIC values using AGSP criteria 2023. All beta-lactamase producing isolates are penicillin resistant.

Supplementary Table 2

	Raw	Corrected
**Singletons (n = 1)**	987	878
**Pairs (n = 2)**	172	151
**Isolates paired**	344	302
**Clusters (n ≥ 3)**	259	233
**Isolates clustered**	4569	4720
**Median cluster size**	6	6
**Mean cluster size**	17.6	20.2
**Smallest cluster (isolates)**	3	3
**Largest cluster (isolates)**	642	709
**Median cluster duration (weeks)**	25	25.7
**Mean cluster duration (weeks)**	35.6	43.6
**Minimum cluster duration (weeks)**	1	1
**Maximum cluster duration (weeks)**	227.9	231.9

Summary statistics for the hierarchical clustering analysis using raw pairwise allelic differences compared to corrected the pairwise allelic differences for the temporal distance between isolates.

Supplementary Table 3

Cluster	Mean Pairwise SNP distances
1003	11.0103
1020	14.4889
1030	7.18367
1034	9.61778
1040	6.71875
1043	12.5306
1045	8.66667
1067	10.679
1079	8.20408
1091	4.32653
1094	7.95351
1102	3.72222
1104	8.48
1105	6.48443
1111	7.6875
1126	8.34903
1127	9.7551
1132	6.8
1135	8.48521
1152	3.46939
1160	4.82
1164	6.6782
1177	4.15052
1187	2.34375
1206	5.55102
1213	6.8
14	12.8
146	18.4048
15	31.1513
155	17.3194
180	25.713
22	8.4
230	12.1552
231	11.9055
26	23.6133
268	7.52
277	19.1446
33	18.843
34	12.6875
38	22.375
451	15.5382
50	18.2112
502	21.5395
551	7.40828
554	12.0741
556	12.1509
557	14.9779
558	12.2699
560	10.011
562	8.72533
567	10.4152
570	9.5
574	13.6864
575	7.56233
579	6.57143
605	6.48
614	11.7917
616	8
625	9.68
629	5.55556
649	8.08
657	8.73373
661	6.72222
666	10.0306
681	14.2012
687	6
692	10.355
718	4.84722
740	3.15625
750	12.4938
761	9.6
773	8.42
780	5.59184
791	6.38889
818	9.95366
840	11.9141
844	8.40657
846	4.72222
848	9.36
852	6.04938
859	3.38889
860	6.33333
879	10.655
883	8.08
898	8.07699
926	4.88889
938	10.7778
958	5.72
960	8.06803
966	5.92
967	6.84024
980	11.1301
983	5.28
989	8.11111
991	3.14844
994	9.22222
995	12.1926

The mean pairwise SNP distance for all genomes within each cluster used in Supplementary Figure 15. Pairwise SNP distances were calculated using snp-dists (v1) with the Gubbins filtered SNP alignment as input.

Supplementary Analysis 1:

Applying our cgMLST clustering method to another dataset

All supplementary analyses and code can be found can be found in the GitHub for this manuscript (https://github.com/mtaouk/Neisseria_gonorrhoeae_transmission_Australia/Supplementary_analyses).

490 *N. gonorrhoeae* genomes were made available through De Silva et al. 2016 (accessions can be found in https://github.com/mtaouk/Neisseria_gonorrhoeae_transmission_Australia/blob/main/Supplementary_analyses/cgMLST_method/SraRunTable.csv) (1). These genomes have similar level metadata to our study (within-individual, within-site). We applied the same cgMLST scheme, allele calling and refining methods as described in our study to assess the generalisability of this method. After performing allele calling on the De Silva genomes, we refined the cgMLST schema to 1,506 genes were present in 95% of isolates and used these genes as the schema. This was comparable to our refined scheme of 1,495 genes. 1,476 of the genes retained in the De Silva 95% schema were included in our refined scheme. Here we show the overall distribution of pairwise allelic differences across the De Silva genomes:

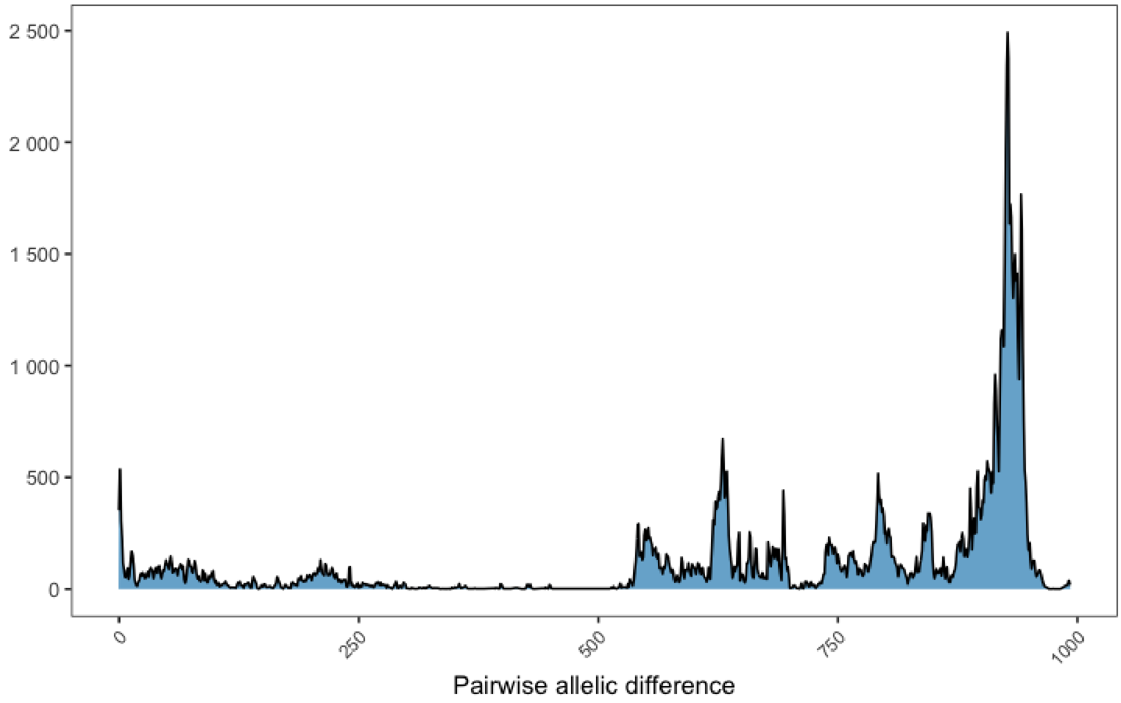

To determine an appropriate clustering threshold for the De Silva dataset we plotted the distribution of pairwise allelic differences of the within-individual pairs and within-site pairs of genomes, applying the same principle as

for selecting the clustering threshold in our own dataset. All within-site pairs of genomes were between 0 and 3 allelic differences apart. The within-individual pairs of genomes were between 0 and 944 allelic differences apart. However, the majority of within-individual pairs fell within the 0 to 7 range. This is comparable to our own results, where we also saw co-infections amongst our within-individual pairs, representing the high pairwise allelic differences.

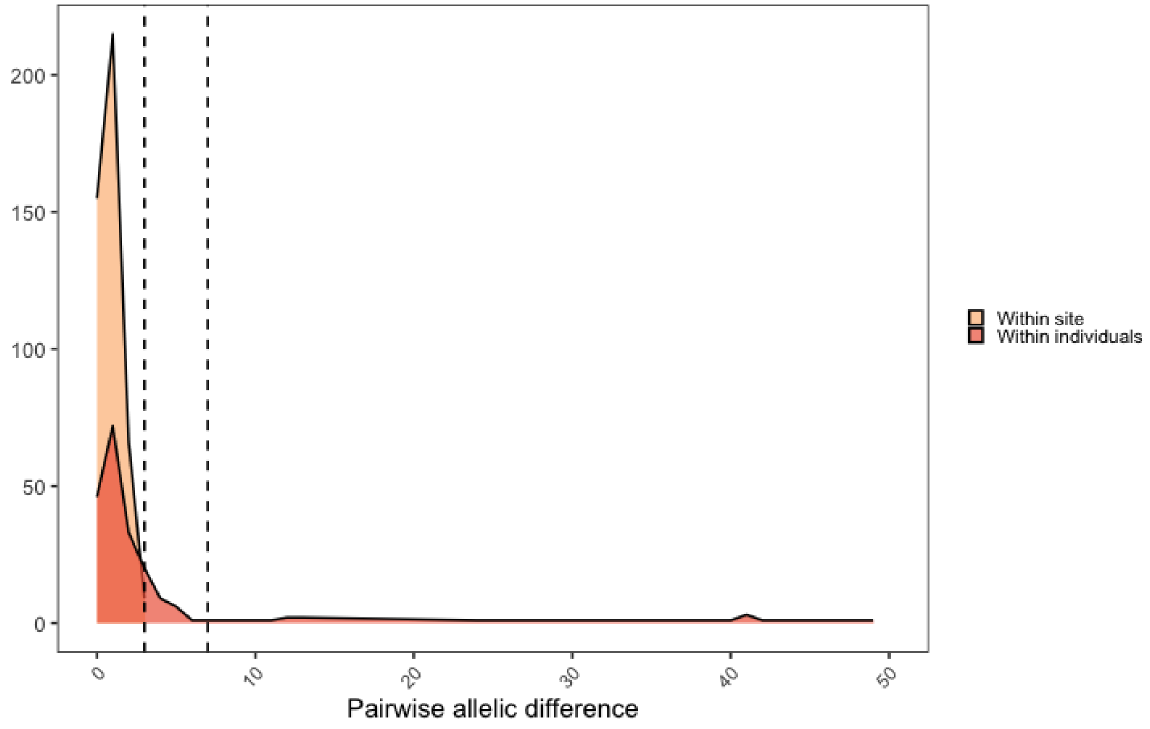

The majority of the within-individual isolates fell below 7 pairwise allelic differences ,suggesting that it would be an appropriate threshold to use in this case. While the datasets and scheme are not identical, the same methods can be applied to any *N. gonorrhoeae* dataset to investigate transmission and a threshold can be applied based on available calibration isolates as done here, or a similar threshold of 7 pairwise allelic differences could be generalizable to other studies if no calibration isolates are available.

When applying the threshold of 7 allelic differences as a threshold for single linkage hierarchical clustering to defining genomic transmission clusters 32 singletons (n = 1), 70 pairs (n = 2) and 46 clusters (n ≥ 3) were identified.

Supplementary Analysis 2:

Comparing cgMLST to SNP based approaches

The trimmed paired end reads were aligned to the NCCP11945 reference genome using Snippy (v4.3.5), requiring a minimum of ten supporting reads and a variant frequency of 0.9 or greater. Recombination filtering was performed using Gubbins (v2.4.1) with default settings and the full Snippy pseudoalignments as input (2). Following Gubbins, a core SNP alignment was generated using snp-sites (v1) and the Gubbins filtered alignment as input with the -c flag (3). The number of constant sites from the whole genome pseudoalignment was also calculated using snp-sites with the -C flag (v1). A ML phylogenetic tree was inferred using IQ-tree (v2.0.3), with the best-fitting nucleotide substitution model chosen based on the lowest BIC and the number of constant sites specified (4). Molecular dating of ancestral events was performed using the least-squares dating (LSD) software (v0.3), with the whole dataset maximum likelihood phylogeny generated here used an input (5).

Using this method, the core SNP alignment consisted of 8,842 core sites (polymorphic/variants that are present in all samples). While using a core SNP alignment can be used to build a high-resolution phylogeny in many cases and has been the gold standard approach in bacterial phylogenetics for the past decade, when applied to a large and diverse dataset as in this case, it can result in a shrinking of informative sites, and a less resolved phylogeny. N. gonorrhoeae is a very diverse species with much recombination, therefor the number of sites conserved across various samples is smaller. For example, across the full whole genome pseudoalignment, there is a minimum of 6% N sites (134,866 bp) for any genome. As these N sites will be dispersed mostly randomly across the genome, the chances of any site having at least one N in at least one sample is high and means that site will be excluded from the core SNP alignment, even if it is informative to the phylogeny. One way to increase the number of core SNP sites is to remove genomes with a high proportion of N sites from the alignment and analysis. In this case, 1,184 genomes have more than 10% N sites. Excluding these would remove 20% of isolates from the phylogeny and clustering analysis – decreasing our sampling proportion and introducing a high level of uncertainty into our clustering. As a result, we opted to use a cgMLST method, a much more permissive way of comparing relatedness across very diverse genomes in a large dataset.

The concern that using a strict core results in isolates potentially being classified as more closely related than they would be stems from the principle of using a static SNP threshold to define transmission. For example, a common SNP threshold of 10 SNPs would result in much more permissive clustering using a core SNP alignment of one

hundred sites compared to using the same threshold on a larger core of thousands, as the threshold represents a fraction of the total sites. We acknowledge that inferring transmission events could benefit from clade- or cluster-specific alignments. By generating alignments specific to a clade or core genome group and mapping reads to a genetically closer reference genome, it would be possible to obtain more genetically informative insights compared to what is obtained based on cgMLST.

Regardless, we have generated a recombination filtered core genome SNP ML phylogeny:

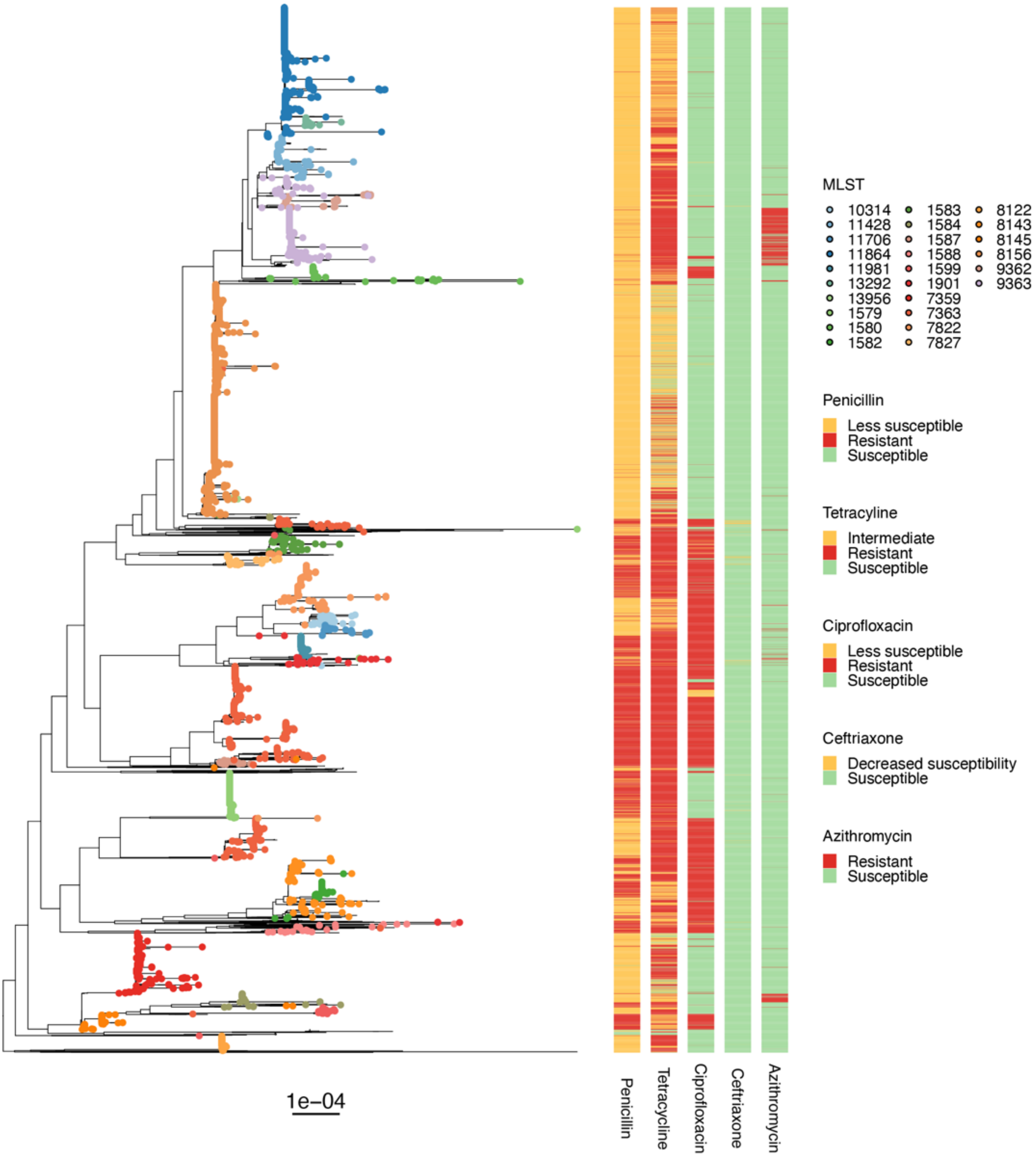

We can see that the overall population structure is mostly conserved, however the resolution at recent evolutionary events is much reduced when compared to the concatenated cgMLST phylogeny (Supplementary Fig. 8). Additionally, we generated a timed phylogeny using LSD and the ML SNP tree from above. This resulted in a phylogeny with a tMRCA of 1845.73 compared to the cgMLST tree root of 1873.13 (Supplementary Fig. 9).

Additionally, we calculated the pairwise SNP distances across the dataset from the recombination filtered core SNP alignment using snp-dists (v1). The median pairwise SNP distance between individuals was 122 (range 0 to 769), within individuals was 0 (range 0 to 211), within sites was 0 (range 0 to 0) and in paired couples was 0 (range 0 to 0). We find that using a strict core SNP alignment of 8,842 sites, decreases the resolution of relatedness between any isolates and decreases the utility of our calibration isolates in determining a threshold, as the maximum pairwise SNP distance is 0.

Supplementary Analysis 3:

GEE odds ratio model using continuous MIC variables

To assess variables associated with cluster persistence, we applied a multivariable logistic regression model via generalised estimating equation (GEE) to calculate adjusted odds ratios using an independence model with geepack (v1.3.9). The following specified variables were included in the models: age group, sex, size of transmission cluster, phenotypic resistance to penicillin, phenotypic resistance to tetracycline, phenotypic resistance to ciprofloxacin, phenotypic resistance or decreased susceptibility to ceftriaxone and phenotypic resistance to azithromycin. For sex, an 'unknown' category was included to accommodate missing data, with all other categories having complete data. In this supplementary analysis we used continuous MIC values for the phenotypic antimicrobial susceptibility profiles. For the phenotypic antimicrobial susceptibility profiles, each breakpoint has been assigned a value from 1 to 11 based on the antibiotic.

```
 Coefficients:
               Estimate   Std.err   Wald Pr(>|W|)
(Intercept)    4.928596  1.617803  9.281  0.00232 **
SexM          -1.275334  0.347233 13.490  0.00024 ***
SexOther      -2.147489  0.721981  8.847  0.00294 **
AgeGroup20-29 -0.091852  0.345161  0.071  0.79015
AgeGroup30-39 -0.258350  0.323177  0.639  0.42405
AgeGroup40-49 -0.452894  0.419387  1.166  0.28019
AgeGroup50-59 -0.468398  0.499611  0.879  0.34849
AgeGroup60-69 -0.547614  0.609648  0.807  0.36905
AgeGroup70-79  0.018856  0.699239  0.001  0.97849
Size           0.017994  0.006914  6.774  0.00925 **
PEN           -0.245739  0.376097  0.427  0.51350
TET           -0.211504  0.297985  0.504  0.47784
CTRIX         -0.056818  0.629740  0.008  0.92811
CIPRO         -0.143813  0.162365  0.785  0.37576
AZITH         -0.959885  0.353599  7.369  0.00664 **
---
Signif. codes:  0 '***' 0.001 '**' 0.01 '*' 0.05 '.' 0.1 ' ' 1

Correlation structure = independence
Estimated Scale Parameters:

            Estimate Std.err
(Intercept)   0.6458    2.57
Number of clusters:   31  Maximum cluster size: 709
```

```
# A tibble: 15 × 7
   term          estimate std.error statistic  p.value conf.low conf.high
   <chr>            <dbl>     <dbl>     <dbl>    <dbl>    <dbl>     <dbl>
 1 (Intercept)    138.      1.62      9.28     0.00232   5.80    3293.
 2 SexM             0.279   0.347    13.5      0.000240  0.141      0.552
 3 SexOther         0.117   0.722     8.85     0.00294   0.0284     0.481
 4 AgeGroup20-29    0.912   0.345     0.0708   0.790     0.464      1.79
 5 AgeGroup30-39    0.772   0.323     0.639    0.424     0.410      1.46
 6 AgeGroup40-49    0.636   0.419     1.17     0.280     0.279      1.45
 7 AgeGroup50-59    0.626   0.500     0.879    0.348     0.235      1.67
 8 AgeGroup60-69    0.578   0.610     0.807    0.369     0.175      1.91
 9 AgeGroup70-79    1.02    0.699     0.000727 0.978     0.259      4.01
10 Size             1.02    0.00691   6.77     0.00925   1.00       1.03
11 PEN              0.782   0.376     0.427    0.514     0.374      1.63
12 TET              0.809   0.298     0.504    0.478     0.451      1.45
13 CTRIX            0.945   0.630     0.00814  0.928     0.275      3.25
14 CIPRO            0.866   0.162     0.785    0.376     0.630      1.19
15 AZITH            0.383   0.354     7.37     0.00664   0.191      0.766
```

We see that sex and size of cluster are still associated with persistence of clusters.

GEE odds ratio model where intermediate isolates are grouped with resistant isolates

The following specified variables were included in the models: age group, sex, size of transmission cluster, phenotypic resistance to penicillin, phenotypic resistance to tetracycline, phenotypic resistance to ciprofloxacin, phenotypic resistance or decreased susceptibility to ceftriaxone and phenotypic resistance to azithromycin. For sex, an 'unknown' category was included to accommodate missing data, with all other categories having complete data. In this supplementary analysis isolates were grouped binarily as either phenotypically resistant/less susceptible/decreased susceptibility or susceptible.

```
 Coefficients:
                  Estimate Std.err  Wald Pr(>|W|)
(Intercept)        -3.8487  1.8957  4.12   0.0423 *
SexM               -1.0740  0.5143  4.36   0.0368 *
SexOther/Unknown   -1.8078  0.7005  6.66   0.0099 **
AgeGroup20-29      -0.3327  0.3757  0.78   0.3758
AgeGroup30-39      -0.3826  0.3364  1.29   0.2554
AgeGroup40-49      -0.5965  0.4673  1.63   0.2018
AgeGroup50-59      -0.7065  0.4866  2.11   0.1465
AgeGroup60-69      -0.0922  0.6831  0.02   0.8926
AgeGroup70-79      -0.3989  1.3806  0.08   0.7726
Size                0.0136  0.0060  5.15   0.0232 *
PENSUS              2.5868  0.9328  7.69   0.0056 **
TETSUS              0.8578  0.6361  1.82   0.1775
CTRIXSUS           -1.7719  1.1047  2.57   0.1087
CIPROSUS            1.6711  1.0114  2.73   0.0985 .
AZITHSUS            4.6184  0.9599 23.15  1.5e-06 ***
---
Signif. codes:  0 '***' 0.001 '**' 0.01 '*' 0.05 '.' 0.1 ' ' 1

Correlation structure = independence
Estimated Scale Parameters:

            Estimate Std.err
(Intercept)    0.622   0.513
Number of clusters:   31  Maximum cluster size: 709
```

```
# A tibble: 15 × 7
   term             estimate std.error statistic    p.value  conf.low conf.high
   <chr>               <dbl>     <dbl>     <dbl>      <dbl>     <dbl>     <dbl>
 1 (Intercept)        0.0213   1.90       4.12   0.0423      0.000519     0.875
 2 SexM               0.342    0.514      4.36   0.0368      0.125        0.936
 3 SexOther/Unknown   0.164    0.700      6.66   0.00986     0.0416       0.647
 4 AgeGroup20-29      0.717    0.376      0.784  0.376       0.343        1.50
 5 AgeGroup30-39      0.682    0.336      1.29   0.255       0.353        1.32
 6 AgeGroup40-49      0.551    0.467      1.63   0.202       0.220        1.38
 7 AgeGroup50-59      0.493    0.487      2.11   0.147       0.190        1.28
 8 AgeGroup60-69      0.912    0.683      0.0182 0.893       0.239        3.48
 9 AgeGroup70-79      0.671    1.38       0.0835 0.773       0.0448      10.0
10 Size               1.01     0.00600    5.15   0.0232      1.00         1.03
11 PENSUS            13.3      0.933      7.69   0.00555     2.13        82.7
12 TETSUS             2.36     0.636      1.82   0.178       0.678        8.20
13 CTRIXSUS           0.170    1.10       2.57   0.109       0.0195       1.48
14 CIPROSUS           5.32     1.01       2.73   0.0985      0.733       38.6
15 AZITHSUS         101.       0.960     23.1    0.00000150 15.4        665.
```

Here we see that sex, size of cluster and azithromycin susceptibility are still associated with persistence of clusters. Additionally, susceptibility to penicillin is now associated with persistent clusters. The trends in phenotypic AMR

patterns are consistent across the two models, however we chose to use the binary model as it reflects clinical breakpoints and is simpler to interpret.

References

1. De Silva D, Peters J, Cole K, Cole MJ, Cresswell F, Dean G, et al. Whole-genome sequencing to determine transmission of *Neisseria gonorrhoeae*: an observational study. Lancet Infect Dis. 2016;16(11):1295-303.
2. Croucher NJ, Page AJ, Connor TR, Delaney AJ, Keane JA, Bentley SD, et al. Rapid phylogenetic analysis of large samples of recombinant bacterial whole genome sequences using Gubbins. Nucleic Acids Research. 2014;43(3):e15-e.
3. Page AJ, Taylor B, Delaney AJ, Soares J, Seemann T, Keane JA, et al. SNP-sites: rapid efficient extraction of SNPs from multi-FASTA alignments. Microbial Genomics. 2016;2(4).
4. Nguyen LT, Schmidt HA, von Haeseler A, Minh BQ. IQ-TREE: a fast and effective stochastic algorithm for estimating maximum-likelihood phylogenies. Mol Biol Evol. 2015;32(1):268-74.
5. To T-H, Jung M, Lycett S, Gascuel O. Fast Dating Using Least-Squares Criteria and Algorithms. Systematic Biology. 2015;65(1):82-97.
