# Supplementary material for: Longitudinal genomic analysis of Neisseria gonorrhoeae transmission dynamics in Australia
Source: Nat Commun. 2024 Sep 14;15:8076. doi: 10.1038/s41467-024-52343-0 (PMC11401900; doi:10.1038/s41467-024-52343-0)
Supplement: Supplementary file 3 — Description of Additional Supplementary Files [file 41467_2024_52343_MOESM3_ESM.pdf]

Description of Additional Supplementary Files

File Name: Supplementary Data 1
Description: All patient metadata, phenotypic and genotypic data, and accessions.
